# “What support?”: A qualitative study on social support for Asian American victims of racism during the COVID-19 pandemic

**DOI:** 10.3389/fpubh.2022.961215

**Published:** 2022-10-19

**Authors:** Sherry C. Wang, Bianca Marie C. Santos

**Affiliations:** Department of Counseling Psychology, Santa Clara University, Santa Clara, CA, United States

**Keywords:** Asian American, qualitative research, anti-Asian racism, social support, COVID-19

## Abstract

Since the COVID-19 pandemic, anti-Asian racism has surged, yet little is known about Asian Americans' experiences of social support. Therefore, we designed a qualitative, intrinsic, revelatory case study to examine the nature and quality of social support for Asian Americans during the first 6 months of the pandemic. Our sample consisted of 193 Asian Americans (from over 32 U.S. states) disclosing their experiences of inadequate social support. They described their support network as (1) Being unable to relate, (2) Encouraging their silence, (3) Minimizing anti-Asian racism, (4) Denying anti-Asian racism, and (5) Victim-blaming. Regarding our participants' recommendations for increasing social support for Asian Americans, a total of seven recommendations emerged: (1) Legitimize anti-Asian racism, (2) Teach Asian American history, (3) Destigmatize mental health resources to make them accessible for Asian American families (4) Promote bystander intervention trainings, (5) Build solidarity with and beyond Asian Americans to dismantle racism, (6) Increase media attention on anti-Asian racism, and (7) Elect political leaders who will advocate for Asian Americans. Altogether, our findings underscore the need for systemic forms of advocacy to combat anti-Asian racism, and shed light on the injurious nature of social support for Asian American victims of racism.

## Introduction

“[We need] non-Asians to acknowledge that anti-Asian racism and prejudice is not a new thing that happened with coronavirus. And, [we need] Asians to realize this too and stop minimizing the struggles that their parents, grandparents, and friends face just because they have not. Then, [we need to] give Asian Americans more resources to learn about racism; and [we need] Asian American history in order to educate the public. [We also need to] give this education to others as well (along with Black, Native, Hispanic [history], just [as long as it is not] White supremacist centered history).”

The quote above is based on a participant's recommendations for improving Asian American social support due to the influx of anti-Asian racism during COVID-19. Since the COVID-19 pandemic emerged at the beginning of 2020, overt discrimination toward Asian Americans has been recognized across the U.S. (1–3) prompting increased documentation of anti-Asian racist incidents (4, 5). However, extant literature on “Asian American” and “racism” emphasize individual-level processes (e.g., coping styles, self-esteem, racial identity) with less attention paid to contextual, community, and systemic interventions needed to combat racism. There is little focus on the contexts and people surrounding Asian Americans to address opportunities for meaningful, systemic change. Consequently, the research literature on Asian Americans, racism, and social support, have primarily focused on individual-level points of intervention, with less attention paid to the social support network of Asian Americans, much less their inadequacies in supporting Asian Americans.

There is a small and burgeoning body of research on Asian Americans, racial discrimination, and social support. Social support is generally defined as aid and assistance provided in relationships and interpersonal transactions that is intended to be helpful (6). As a whole, research on Asian Americans and social support have highlighted their underutilization of formal and informal sources of supports. Data on Asian American help-seeking have found that they are half as likely as than White American counterparts to mention mental health problems to friends or relatives, and to also use health services, such as seeking mental health care services or even self-help groups, at a lower rate (36 vs. 56%) (7). In fact, much of the literature shows that Asian Americans tend to rely on informal services preferring spiritual advisors, internet support groups, self-help groups, over formal services, such as from psychologists, family doctors, or other healthcare professionals (8). Particularly in the context of racial discrimination, research has shown that Asian Americans tend to turn to informal sources of social support rather than professional services (9–11). The lack of formal help-seeking among Asian Americans have been attributed to a range of reasons, from mental health stigma in the Asian American community (12), to lack of culturally and linguistically accessible healthcare services (13), to cultural stereotypes such as the model minority myth (MMM), which positions Asian Americans as being a problem-free monolithic group known for being successful, hardworking, and with “cultural values” that make them a “model minority” (14).

Regarding the role of informal social support for Asian Americans, the findings have also been mixed. Measurement issues may be a key contributor of the inconsistent findings, because of how social support is conceptualized and measured across studies. As an example, scholars such as Mossakowski and Zhang (15) measured social support based on frequency, such as how frequently peopled talked on the phone or got together with family or friends. They also assessed emotional support based on whether participants felt like they could talk to family or friends about their worries, and their sense of family and friends being able to them to help with serious problems. Other quantitative studies (16–18) have also utilized scales or questionnaires to assess participant perception of social support, such as the degree to which they feel emotionally supported and helped by their family members, being able to open up to friends or romantic partners about problems, and being looked out for by neighbors in their local environments. While these aspects of support are important to understand, these studies ultimately do not allow participants to determine what they subjectively determine to be forms of social support to them; additionally, it is unknown how salient social support may be from one person to another. That is, the social support identified by researchers may or may not be relevant or even salient to some peoples' lived experiences. Additionally, while some researchers study family and friends separately, others make further delineations by distinguishing romantic partners from other family members, or by studying all of these different groups. Finally, given that the existing body of scholarship is primarily quantitative, the research designs inherently rely on researcher operationalization and identification of constructs and instruments to assess social support. In these ways, participants do not have the opportunity to share what is uniquely and subjectively considered to be their forms of support, nor are they able to provide feedback on the quality of the support that they receive (and the lack thereof).

In the context of racial discrimination, researchers have largely treated social support as a form of coping. That is, research on the social support of Asian American adults experiencing racism has been rooted in the premise of social support being a buffer. Social support is often tested for its effects in moderating the effect of racism on psychological distress and adverse mental health symptoms (e.g., depressive symptoms, anxiety, loneliness, trait anger). However, the buffering effect of social support has been mixed and nuanced across different networks of support. With regard to social support from family members, Chae et al. (19) found that family support buffers the effects of racism for those who experience lower rather than higher levels of discrimination. Their work underscores the importance of family for those who experience lesser discrimination. In contrast, however, Wei et al.'s (20) work found that family support is not particularly helpful and that they fail to protect Asian Americans from the detrimental effects of racial discrimination. Adding to this complexity is Mossakowski and Zhang's study examining family and peer support (15), in which they found that only certain kinds of family support buffer the psychological distress of Asian Americans (i.e., emotional support in the form of being able to rely on family for serious problems), but that other kinds of family support (i.e., frequency of talking about worries to their family) were not helpful. Because these scholars also incorporated peer support in their study, they found that peer support did not protect Asian Americans from psychological distress. That peer support is less helpful than family support has been contested by the findings of Singh et al. (21) when they found that friend support and not family support moderated the association between Asian American experiences of discrimination and psychological distress. These findings paint a mixed and nuanced picture regarding the role of family and peer support for Asian American victims of racism.

The supportive role of spouses and romantic partners has also been mixed. For the most part, spousal support has not been shown to protect Asian Americans from the effects of discrimination. That is, Rollock and Lui (18) found that although spousal support buffered stress that is related to general unfair treatment, partner support did not offer a protective role against racial discrimination. Thus, it seems that partner support is useful only within the context of overall injustice experiences, but not when it pertains to racial discrimination. Adding to the nuances is work by Chung and Epstein (22), in which the researchers found that not only did spousal support fail to buffer the effects of racial discrimination on distress, but spousal strain actually heightens the association between racial discrimination and psychological distress. These findings suggest that the role of spouses may be more detrimental than helpful in the context of Asian American racial discrimination.

It would seem then, that the social support literature captures complexities and richness in terms of the role of support systems for those experiencing racial discrimination. In fact, studies examining partner and family support have shown that they may actually contribute less to the wellbeing of Asian Americans; and instead, contribute to greater distress. Kwon's study (23) examined the mediating and moderating effect of family and spousal relations on perceived discrimination and psychological distress and found that both family cohesion and spouse/partner support did not provide a buffering effect; furthermore, negative family interactions can actually worsen things, such that family conflict exacerbates the effects of discrimination. Taken together, these findings underscore the need to understand the impact and quality of social support rather than assuming positive intent. Even more broadly, there is a need to understand how Asian Americans experience social support, and to learn about their interpretations of social support in the context of racial discrimination.

Despite the need to understand Asian American experiences of support amidst racial discrimination, research in this area has largely lumped together the different sources of social support, whether it is family, partner/spousal, friend, colleague support. Even outside of the focus of racial discrimination, scholars have found statistically insignificant findings when testing the stress-buffering effect of general social support among Asian Americans, such as among Indian American (24), Afghan American (25), and Filipino American (26) populations. As such, more scholarship is needed on Asian American social support to explain the nuanced findings.

In recognizing these limitations, our study builds on the existing scholarship on Asian American social support and racial discrimination. Our goal was to examine experiences of inadequate social support for Asian Americans, based on real-life experiences of when they sought and received social support in the aftermath of COVID-19-related anti-Asian racism. To do this, we designed a qualitative research study using open-ended questions to inquire about their social support experiences. We did not make any a priori assumptions or pre-determined definitions of social support. Our purpose was to understand how participants interpreted the quality of their social support (or lack thereof) and to solicit recommendations for providing better support. By examining the impact rather than the intent of social support for Asian Americans, our research study allowed for the possibility of understanding how social support fails Asian American victims of racism, and how it can be improved.

## Methods

We designed this study to be an intrinsic qualitative case study (27), which is a research design intended to capture a phenomenon in a single case that is bounded by time and space. The phenomenon for this study was social support, specifically the inadequate social support received by Asian Americans during a time of intensified of anti-Asian racism. In terms of time, our study was bound to the first 6 months of the COVID-19 pandemic, from April to October 2020, during a period of increasing anti-Asian violence. Because case studies are also bound by space, we focused exclusively on the experiences of Asian Americans in the U.S. context. Given the timing of our study, particularly during the onset of the pandemic the study became a revelatory, intrinsic case study due to the uniqueness of the contemporary event.

### Positionality/researcher stance

Our research team was comprised of the first and second authors, both of whom identify as Asian American and Asian international, respectively. Like our participants, we were also affected by the surge of anti-Asian racism accompanying the COVID-19 pandemic. That is, we were also part of the revelatory nature of our research design. At a time in which the country was going through social distancing and sheltering-in-place, our research team was also living the same kinds of experiences as our participants. For example, our study was conducted entirely online, with all of our research meetings held virtually through Zoom or *via* phone calls. Consequently, in reflecting on our biases, we were vigilant about how we might over-identify with our participants' experiences.

### Participants and sampling

To be eligible for this study, participants had to be at least 18 years of age and self-identify as “Asian American.” English proficiency was not an exclusion criterion; however, the study was only available in English. All of the data were based on self-report. We relied exclusively on online methods, given that our data was collected during a time of sheltering-in-place. We began recruitment through Asian American organizations nationwide using the Google search engine, contacting 102 organizations and following up with them twice. Additionally, we contacted active social media accounts that addressed topics pertaining to diversity, minority mental health, activism, and Asian American and other POC communities, in total, contacting 12 Instagram influencers and nine Facebook groups, to ask for assistance in disseminating our study to their audiences. Additionally, the first author promoted the study in webinars and presentations that centered on anti-Asian racism in the COVID-19 pandemic. Due to the sensitive nature of the survey questions, we did not collect any additional demographic information from the participants. No financial compensation was offered for completing the survey.

### Data collection and procedure

The current investigation is part of a larger project on anti-Asian racism during COVID-19 [for more information about methodology, see (27)]. Data for this study were collected online through open-ended questions in the Qualtrics platform. For this manuscript, the responses we analyzed had to do with participants' experiences of support during the COVID-19 pandemic. Specifically, we asked whether participants were able to find support after experiencing anti-Asian racism. Additionally, we followed up with, “If so, from whom/where? If not, what kind of support was missing?” We also asked general questions like, “Have you felt supported or not supported as an Asian American during the COVID-19 pandemic?”), as well as their hopes for support (“If you have not felt supported, or if you would like more support, what resources do you wish exist for the Asian American community?”). Finally, we asked for their recommendations for empowering the Asian American community (“What could help the Asian American community better respond to anti-Asian racism?” and “How could the larger society, especially non-Asian Americans, help to empower the Asian American community?”). All protocols and materials were approved by the Institutional Review Board at Santa Clara University.

### Data analysis

We utilized the explanation-building technique within a case study design to explain “how” or “why” a phenomenon occurred (28). Yin (28) has described this analytic technique as a type of pattern-matching that focuses on building explanations. That is, because our study phenomenon had to do with our participants' experiences of social support, we analyzed the data to examine how respondents defined, explained, and experienced social support and its consequences. We also followed Braun and Clarke's six stages of thematic analysis (29), which follows a process of building familiarity with the data, reading the answers by participant, by question, identifying initial emerging themes, and then corroborating the themes through an iterative process of revisiting each response for deeper immersion in the data. We chose quotes that would most powerfully convey our participants' experiences. Regarding coding disagreements, we discussed them until consensus was reached, whenever they emerged. Although power dynamics are relevant in any research team, we specifically devoted substantial time and attention to discussing and acknowledging the power differentials in our research team as well as taking notes in reflexive journals and memos.

### Data validation

To establish the credibility of this study, we used key strategies that have been implemented in qualitative research to enhance methodological rigor: triangulation and researcher reflexivity (30). Our data was triangulated across a wide range of participant responses given our large sample size and the rich findings that emerged. We also engaged in researcher reflexivity throughout the data analysis by reflecting on our biases and assumptions through the form of self-reflective journaling.

## Results

A total of 201 participants completed the study, but due to missing data, only 193 participant data were included for data analysis. Participant ages ranged from 18 to 65+ years of age. More than two-thirds of the participants (69%) identified as younger than 34 years (35% were 18-24 and 34% were 25–34), followed by the 35–44 age group (18%). Approximately 9% reported being in the 45–54 age range and the category of 55–64, and 65 or older each reflected 1% of the data. Participants resided across 32 states, District of Columbia, and Puerto Rico or lived outside of the U.S. at the time of survey completion. A majority of respondents were from California (40%) and New York (12.5%).

Participants experienced a wide range of responses from their support systems. In describing the ways that they were not supported, a total of five themes emerged, ranging from being minimally harmful to most harmful. These were: (1) Being unable to relate to anti-Asian racism, (2) Encouraging silence, (3) Minimizing anti-Asian racism, (4) Denying anti-Asian racism, and (5) Victim-blaming. Additionally, our participants identified that their perpetrators were White partners/spouses, White peers, family members, Asian American friends, non-Asian People of Color friends, as well as unspecified “others.” The latter group included people such as their colleagues, neighbors, and their online network. A visual overview of these findings can be found in Table 1. In the sections below, we discuss each of these themes before presenting participants' recommendations for how to better support victims of anti-Asian racism.

**Table 1:** Experiences of inadequate social support by type and people.

	**White peers**	**White partners/spouses**	**Family**	**Asian friends**	**Non-Asian POC friends**	**Unspecified “others”**
Being unable to relate	X	X	X			X
Encouraging silence			X	X		
Minimizing anti-Asian racism	X		X	X	X	X
Denying anti-Asian racism	X	X			X	X
Victim-blaming	X		X		X	X

### Being unable to relate

Participants described how they needed, craved, and hoped to receive support in the aftermath of racist experiences; yet, they were let-down by the responses of well-meaning and well-intentioned people. They specifically named White partners/spouses and White friends as people in their support network who were unable to understand their experiences of racism. White partners/spouses, in particular, were unable to relate to our participants' experience, which exacerbated the loneliness they already felt. That is, not only did participants already feel alone from their experiences of being victims of anti-Asian racism; the subsequent experience of having no one understand their experience exacerbated their sense of isolation.

I talked about this incident with my spouse, who is a white European immigrant. He listens empathically but does not share my fears… I do not feel that any one close to me truly understands how distressing it is to me.

Participants acknowledged the good intentions of White friends, colleagues, and partners trying to support them, by adding caveats and background information, such as “he's very understanding but has trouble relating…” or “I did get some support from him… but… it made him uncomfortable.” In another scenario, when a participant described having the support of White friends during a time of social distancing, the participant described them as being “unable to truly understand why I am so paranoid about going outside even to buy essential goods.” Additionally, people in positions of authority, such as professors, bosses, and supervisors were described by our participants as being unhelpful given that “none [of them] were able to personally relate” to anti-Asian racism.

Our participants also identified the limitations of family members, specifically Asian American parents and siblings, regarding their inability to offer emotional support:

Asian parents aren't the most supportive. At least my parents aren't. There's no hand holding, no hugs, no “are you alright?” You're supposed to be smarter than everyone and not shed a tear.

One participant shared, “I wish my own family would emotionally support me more” while another explained that they do not talk about racism with their mother or siblings because “we don't talk about our feelings with each other.” Thus, participants also felt that their family members were unable to relate to them since their families were described as being incapable of providing emotional support.

### Encouraging silence

This theme refers to the messages relayed to our participants for them to be quiet, stay silent, and avoid drawing additional attention to anti-Asian racism. Below, a participant shares their experience of their family's intergenerational transmission of racial trauma, referring to the salience of the Japanese internment camps in their present-day experiences. They describe how their grandparents and parents have been socialized to be silent and to stay under the radar:

I grew up with the terror of internment camps looming in our family history. My grandparents refused to give my mom and aunts/uncle Japanese names, they never spoke Japanese again (therefore the language stopped with my grandparent's generation) they were afraid that it happened once, it could happen again. Even to this day my mom, and aunts/uncle are very scared of speaking up or making waves.

Participants especially emphasized their parents' role in socializing them to be silent, such that they were “taught to lay low, work hard, don't ruffle feathers, and assimilate.” Another participant stated: “my parents taught me, scratch that, they basically threatened me to be quiet. Be a good kid and not speak. To never cause a scene and to never start something controversial.” One participant stated, “I was taught by my parents to just put my head down and keep going—that talking about it was bringing unnecessary attention (causing drama).” As such, our participants received implicit and explicit messages from family members to maintain a culture of silence about anti-Asian racism.

Beyond the family, our participants also shared their disappointment with Asian American friends and peers for maintaining this same culture of silence.

I have not [felt supported]. I'm very vocal about race issues with the Asian community and most of my peers (majority Asian) choose to shun it out and avoid the topic… Our community has not spoken up, shared our frustration, and made any noise on the issue. I'm deeply disappointed in my Asian peers who kept quiet. It's hard enough for the vocal ones; but, to not even have the support of our own people makes it that much harder.

Participants therefore reported feeling disappointed in, dismayed by, and even angry at fellow Asian Americans for their silence. They described feelings of isolation, with one participant sharing, “I wish, in general, my Asian American friends were more open in talking about their experiences.” The same participant added, “I just want another Asian American to completely share their feelings with me so I don't feel alone… an Asian American adult ally who I could share experiences with.”

### Minimizing anti-Asian racism

Participants described instances in which their support system responded to their experiences of racism by either downplaying the severity of the incident, minimizing the participants' distress, or both. Participants described these to be normative experiences, such that “most of the White people I know have been passive or even told me to get over it.” Even in relaying these experiences to their parents, participants received similar messages that they needed to just “get over it.” Below, a participant describes not only the pain of being victimized by racism, but also the pain of having their parents downplay their distress.

I also wish my parents would have been more openly understanding of how hurt I felt whenever people were racist/discriminatory to me. Too often, my parents said that I should “brush it off.”

These quotes capture our participants' experiences of having their racial trauma trivialized. In other situations, participants noted that they could not even find spaces to talk about their racial trauma. Put succinctly, one person shared: “I wasn't able to find support. Even if I tried, people around me talked over it, claiming their experience is worse.”

Participants described the pressure of needing to prove the severity of anti-Asian racism to be able to gain the empathy and support of others. Additionally, they received messages that anti-Asian racism is “not as bad” when compared to the racism of other People of Color. That is, Asian American racism was considered to be acceptable because of how tolerable microaggressions were against Asian Americans, and how frequently they were “normalized as OK behaviors” in the larger society. One participant added that Asian Americans internalize these beliefs as well: “Asian American racism is often minimized because we usually get the ‘good stereotypes.' Even though we experience racism fairly regularly in this country, we tend to brush it off because we know that it is not as concerning to people and because the Asian American community in general, does not like to talk about big issues like this.” As such, the minimization of anti-Asian racism seemingly came from everywhere: from within the Asian American community, to non-Asian People of Color, to White people.

### Denying anti-Asian racism

Participants described instances in which their support network invalidated their victimization, altogether. One respondent recalled disclosing a racist incident to their White European immigrant husband, who subsequently justified the victimization by suggesting, “maybe he [the perpetrator] thought you were pretty.” The participant added that “[my husband] thought I was exaggerating.” In another situation, a participant described having multiple people in their life invalidate their experience of racism. Specifically, “many White friends said I was just paranoid and making a big deal out of this. They said I was just trying to play victim and spreading paranoia.” Therefore, White partners as well as White friends seemingly responded in ways that not only dismissed the distress of the participants, but they created greater harm by making light of the situation and telling the victim that they imagined the incident to be more severe than it was.

Participants described the ways in which non-Asian People of Color, namely Black and brown people, invalidated their experiences of racism by refusing refer to Asian Americans as People of Color and consequently denying the existence of anti-Asian racism altogether. One participant shared: “[Anti-Asian racism has] been dismissed so hard by other minorities, it is extremely frustrating (such as being called Not a POC, racism against Asians isn't real, and more).” In some circumstances, our participants did not specify who their support system was and as a result, it was unclear who they were referencing when they referred to these unspecified individuals. However, these unspecified others contributed to invalidating our participants' experiences of racism by denying Asian American racism and therefore, denying the distress of those who were victimized by anti-Asian racism.

I told some people and they were always somehow surprised, as though this couldn't possibly happen. But it was and it wasn't surprising at all. It was barely even a step up from the normal amount of discrimination I experience, so it felt, sadly, normal to me. Some people told me that it wasn't happening, I was imagining it, or that it wasn't as bad as I make it out to be.

### Victim-blaming

Our participants described instances in which their support network not only failed to support them, but instead, blamed them for their victimization. In one incident, a participant talked about an instance of having their racist victimization turned against them so that they were treated like a perpetrator instead of a victim. Referring to a White friend, one participant shared, “She… could empathize, but only to a certain extent… asking if I was the initial aggressor or if I did anything to them.” In another situation, a participant described a female White friend as being “dismissive” in that “she blamed the virus on Chinese people and asked me (an Asian American born in the USA) why Chinese people eat weird shit like dogs and bats.” Additionally, with family members, participants also described instances in which members of their support system blamed them by asking them what they did to elicit racism. One participant shared, “I told my mom what had happened. She is the typical Asian mother who finds fault in me for everything and asked if I had done something wrong to provoke it.”

Given that participants also looked to online support during a time of sheltering-in-place and social distancing, they also referred to experiences of being blamed online. Upon reading the writings of an African American blogger, one respondent said:

They claimed that we “had this coming” and “deserve to get hurt for once” due to appropriation of Black culture from K-pop, and general anti-blackness from Asian American communities. It was our turn to die from racism.

Therefore, even though participants shared that they looked to the online community for support in the midst of heightened anti-Asian racism, the virtual space also became an environment that created greater harm. In another situation, a participant shared that they “[saw] a Black community online discuss how they have no reason to support Asian Americans because we've never done anything for them and they blame Asian Americans for racist acts that China has done.” Thus, despite the many benefits of finding community and belonging online, the virtual space was also an environment of scapegoating and blaming Asian American for experiencing racism.

Building on our participants' lived experiences of social support, we asked follow-up questions to solicit their recommendations for better supporting Asian American victims of racism. A total of seven themes emerged, with each theme highlighting systemic-level changes to provide support for Asian Americans. In presenting our findings, our seven themes align with the “most effective solutions in addressing anti-AAPI hate” identified in the Stop AAPI Hate two-year advocacy report (31) and across their three categories of education equity, community solutions, and civil rights. In the category of education equity, our participants recommended the following: (1) Legitimize anti-Asian racism and (2) Teach Asian American history. With regard to community solutions, our participants highlighted the need to (3) Destigmatize mental health resources to make them accessible for Asian American families, (4) Promote bystander intervention trainings, and (5) Build solidarity with and beyond Asian Americans to dismantle racism. Finally, in the category of civil rights expansion, our participants underscored that to do this, our society would need to (6) Increase media attention on anti-Asian racism, and (7) Elect political leaders who will advocate for Asian Americans. Each of these themes are presented below.

### Legitimize anti-Asian racism

Participants believed that acknowledging racism toward Asian Americans is a critical step to advocate for Asian Americans. One participant stated, “They [the larger society] need to understand that Asian Americans experience racism and discrimination.” Participants emphasized the need for Asian Americans to be able to name anti-Asian racism and to be able to conversations about it:

I think talking about it and naming it would help. I was taught by my parents to just put my head down and keep going—that talking about it was bringing unnecessary attention (causing drama). My dad and my brothers still refuse to acknowledge that we are “people of color.” I think we need to be able to talk about these issues openly and validate that this IS real and it IS racism.

Participants further noted the need for everyone to know the long-standing history and nature of anti-Asian racism, so that it is not assumed to emerge solely from COVID-19 stigmatization:

There should be an explicit mention that anti-Asian racism is NOT a new phenomenon that will wax and wane with COVID; instead, COVID and the rhetoric surrounding COVID have given others an outlet for expressing already-situated hate.

Thus, validating the distress of Asian American victims of racism is central for supporting Asian Americans. However, this can only be possible if the larger society recognizes and understands how Asian Americans have been historically and chronically overlooked and excluded from discourse on race and racism. Legitimizing anti-Asian racism would therefore mean that Asian Americans can finally be recognized as victims of racism, rather than be seen as immune and irrelevant to race, racism, and other communities of Color.

Asians have always been excluded in any discussion of race in the US. We were told to stay silent and to deal with the kind of racism we received because it wasn't as bad as it could be. And so, with the rise of hate crimes, it almost felt like we were allowed to express frustration.

Participants emphasized the need for Asian Americans to be included in anti-racism efforts, rather than to minimize and deny anti-Asian racism as a form of oppression that is less valid and less pervasive than racism directed toward other People of Color, specifically, their Black and Brown counterparts.

### Teach Asian American history

Participants highlighted the need to teach Asian American history in mainstream education to educate the general public. One participant implored, “People need to open their eyes to the reality Asians face. It is not a joke and racism should not be taken lightly.” Another participant stated that educational efforts may help with “recognizing the ways in which anti-Asian discrimination is normalized and seen as ‘less bad'.” Participants cited the history of Asian American discrimination and marginalization that dates back to the beginnings of the U.S. as a nation:

I do wish that the recognition of anti-Asian racism was discussed more in our broader society - I feel like the majority of people, including Asians, are not aware of the long history of anti-Asian racism in this country and still hold to outdated beliefs that Asians are not the victims of racism. So, I guess I wish that there was more of a presence of Asian histories and cultures in the way we educate our kids and when we talk about issues in our society.

It is important to note that the recommendation to teach Asian American history meant providing this information to the American public in general as well as to Asian Americans. That is, Asian American history should be taught to all members of society. A participant shared they were embarrassed to admit they did not know about the Chinese Exclusion Act until they took a college course on Asian American experiences in America. Another participant reflected on the absence of Asian Americans in their history classes despite living in an Asian ethnic enclave. Yet another respondent shared that “Asian Americans, by knowing their history deeply, can better understand and use other tactics to respond to racism.” Participants therefore urged for the inclusion of Asian American history in general American education, such as in mandatory K-12 curricula, to facilitate a critical racial consciousness of Asian American racism for all.

### Destigmatize mental health resources to make them accessible for Asian American families

Participants spoke to the need to increase access to mental health resources for Asian Americans. One participant stated, “I wish there were more support groups for Asian Americans to come together, talk, and support each other. And more readily available mental health resources, advice, tips specifically for Asian Americans.” Participants emphasized the stigma surrounding mental health care, naming stigma to be a key barrier in seeking help and support. However, one participant emphasized that supporting Asian Americans meant more than “breaking down the stigma of therapy in the community.” They added that there needs to be “more counseling resources made readily available across generations.” Thus, the stigma of mental health was particularly emphasized as an intergenerational concern. One participant asserted, “Even if you told my parents about the mental health consequences of racism, they won't believe it because they don't believe in therapy or accessing behavioral health services.” Another participant made a similar point about the need for mental health resources for older generations to support younger Asian Americans facing racial discrimination:

In my circle of Asian friends, we always felt that we don't receive much talk about mental health in our families. It seems like a new thing to our parents and relatives that we don't know how to deal with. Sometimes the things we read or hear affects us on a deeper level and makes us dislike being Asian and the desire to cast our culture aside becomes stronger. Having mental health resources to strengthen our resiliency and focus less on the negativity would be helpful.

Participants suggested creating spaces for intergenerational dialogues. A participant suggested the following: “conversations on intergenerational perspectives on mental health could be helpful, especially as it relates to ‘self-care' and older generations considering it to be ‘selfish' [since] the Asian American mode of operating is self-sacrifice.” Participants added that language barriers between generations added to the difficulty of having conversations about mental health. As such, they also called for linguistically-sensitive mental health resources to be able to bridge cultural and generational differences within Asian American families.

### Promote bystander intervention training

Participants recommended widespread training for the general public to know how to intervene as witnesses of a racist incident. A participant who suggested bystander intervention training commented, “In every instance of racism I've experienced, the people who witnessed it never stood up for me. We need people who witness these incidents to speak up and step in to help.” Another participant mentioned feeling shocked and paralyzed by the racism and said, “I needed the White people around me to speak up and they never do.” Participants generally believed that bystander interventions would more effectively address anti-Asian hate incidents than being “keyboard warriors.”

I'd love to have more people trained or even just provided with more information on bystander intervention… using hashtags or whatever on Twitter doesn't make a difference when you're seeing someone being harassed right in front of you.

Participants saw bystander intervention training as taking a proactive stance in addressing racism. One participant thought that it could be a way to mitigate victim-blaming:

I attended a bystander intervention training and I thought that was really helpful. It is more useful to educate non-Asian individuals about their biases than to put the responsibility of fighting anti-Asian racism on the Asian community. It is like asking a sexual assault victim to hold the assaulter's actions accountable. It should not be that way.

Bystander interventions were therefore recommended for combatting anti-Asian racism because of the onus placed on societal responsibility rather than on Asian Americans to stop anti-Asian racism.

### Build cross-ethnic and cross-racial solidarity to dismantle racism

Participants expressed a desire to build community and solidarity across Asian Americans ethnic subgroups, as well as with other racial groups. Within the Asian American community, participants recognized Asian American divisiveness (e.g., “inner-Asian racism,” “Asians against Asians”) and named issues like colorism, the model minority myth, anti-Blackness, “East Asian privilege,” and other within-group conflicts across the Asian American diaspora. One participant recommended that Asian Americans “deeply reflect on our own racist attitudes and behaviors.” To better support Asian Americans, another participant implored:

[We need] a sense of community, a way to foster an Asian American identity that can be separate but also inclusive of different Asian Americans. If there is a greater sense that, for example, a Chinese person and an Indian person and an Indonesian person belong to a larger Asian American community in addition to their own respective communities, then there might be a greater sense of urgency on everyone's part if one group is targeted.

Participants believed that solidarity within the Asian American community could lead to more effective action and advocacy against racism. One participant stated, “Allowing [more] conversation to happen within the [Asian American] community will enable us to hold a stronger position in support of other communities of Color and ultimately work toward eliminating systemic racism.” Thus, participants saw a clear connection with anti-Asian racism and racism directed towards other racial minority groups. Therefore, they recognized the need to strengthen cross-racial solidarity and to speak up against racism against other communities.

In general, participants recognized their “shared fate” and “shared history of oppression” with other People of Color. One participant asserted the need to “connect with other marginalized groups to build bridges so we can support each other.” Another participant echoed this, saying “We as Asian Americans need to stand in solidarity with other communities of color.” Ultimately, participants called attention to the importance of dialogue and building allyship across People of Color as well as white allies. One participant stated, “[Racism] is bigger than all of us, we all need to band together and help one another.” Another participant expressed the process of arriving at their realization for cross-racial allyship and solidarity:

At first, I was resentful to other minority communities and the rest of society for not defending us and expressing outrage to our targeted racism. But then I realized Asians have been quiet when other communities have suffered and not enough of us stood together to support others. I would love to see more campaigning against white supremacy and see all communities banding together to learn how to stand up for one another...

### Increase media attention on anti-Asian racism

Participants were consistent in naming the lack of mainstream media coverage on anti-Asian racism during COVID-19, remarking “the media is not very focused on [anti-Asian racism],” “mainstream media is not covering this topic well” and “I wish the media is not underreporting the racism crime against Asians.” One participant said, “I just wish the media covered our situations more. It genuinely just feels like nobody cares about us.” To address general skepticism of anti-Asian racism, another participant talked about the role of media campaigns “to raise awareness on the issue [anti-Asian racism]: both to increase visibility but also to establish it as a legitimate issue.” Participants recommended less stereotypical portrayals of Asian Americans in the media to potentially combat anti-Asian racism. One participant highlighted the need for Asian American news to be disseminated in national outlets and not just restricted to local Asian American platforms:

It would help if media and mainstream media would acknowledge us and help our stories be heard. Change comes from being educated and aware. We've got to do both and make it seen and heard, to get the message to mass America and not just within our Asian American communities.

Participants therefore looked to mainstream media outlets as a resource for raising their visibility and combatting stereotypical images of Asian Americans. In this way, they placed their hopes on media campaigns to be able to make anti-Asian racism visible and relevant to the U.S. society.

### Elect political leaders who will advocate for Asian Americans

Participants emphasized the role of government officials and community leaders in advocating for Asian Americans as they face racism and stigmatization during COVID-19. Several participants expressed concern and outrage over politicians who “stoke the fires” by using words like “kung flu,” “Chinese virus,” and “China virus.” To counter this, one participant recommended, “Community leaders and lawmakers should make an effort to make visible/audible their support of the Asian community when possible.” Another participant added, “If people in more powerful positions can speak up and admit the racism against Asian Americans, it would help the Asian American community feel less weak and helpless.” Yet another respondent suggested that public leaders should do more than offer verbal condemnation: “Leadership/government should not only call out racism and discrimination (which is performative) but also implement policies, events, campaigns to reduce discrimination for sure...” Participants recognized their ability to hold government officials accountable through their voting power. One participant said, “Asian Americans should be empowered to vote and elect officials that can help with our cause.” Another participant echoed this:

We as Asian Americans cannot afford to sit back and just be satisfied with others bearing the torch for change. We, too, need to be involved and take the most important action that we can: VOTE.

Participants therefore emphasized the need to amplify Asian American political visibility by voting for leaders who will combat Asian American stereotypes, speak out against the existing anti-Asian rhetoric, implement policies and practices that eradicate discrimination, and empower community members to engage in local and national politics.

## Discussion

Our study is the first to qualitatively examine the phenomenon of social support by asking participants about the nature and quality of support received during a time of heightened COVID-19-related anti-Asian racism. Using open-ended questions to inquire about Asian American experiences of social support, our qualitative investigation allowed participants to evaluate the quality of social support that they received in the aftermath of anti-Asian racism. Specifically, our study invited Asian American participants to share how their support systems failed to meet their needs as victims of anti-Asian racism. Additionally, when asked to provide recommendations to improve the social support of Asian Americans, they provided concrete suggestions at the societal and systemic level to underscore the need for collective change to support Asian Americans from anti-Asian racism.

A main takeaway of our findings is that when Asian Americans seek informal support in response to their actual and lived experiences of anti-Asian racism, they receive responses that can lead to greater victimization rather than support. Specifically, our participants provided examples of “support” that left them feeling isolated, misunderstood, gaslit, and even blamed. It is therefore crucial to recognize that well-intentioned social support networks can cause greater harm and victimization, by further wounding those who are already victims of racist marginalization. Our study showed that in the aftermath of anti-Asian racism, it was primarily family members, friends, and partners, who they went to for support; yet, it was also these confidants who offered inadequate support and perpetrated greater harm.

Our descriptive findings help to explain the mixed research findings on Asian American social support and discrimination. In particular, our findings elucidate the reasons why participants may feel even more marginalized upon soliciting support. In the current investigation, our participants' utilization of informal support led to them feeling isolated, misunderstood, invalidated, and even blamed for being victims of racism. Our descriptive findings explain the contradictory findings that have been shown in studies examining racial discrimination and the support-seeking strategies of Asian Americans (32, 33). In studies by Liang et al. (32) and Alvarez and Juang (33), both found that active solicitation of social support was inversely related to psychological distress. Specifically, Liang et al. (32) found that Asian American men's experiences of racism led them to seek more social support, which then led to heightened racism-related stress. Describing these findings as “counterintuitive” and “contrary to [their] expectations,” the authors speculated that the beneficial effects of social support may not be there for helping individuals cope with racism, and that more scholarship is needed to understand accessibility, type, and quality of social support. Similarly, Alvarez and Juang's work (33) found that among Asian American men, support seeking was found to be a mediator with a positive relationship with psychological distress. They, too, concluded that a more refined analysis is needed to understand the nature and quality of support that individuals seek and obtain in response to racism. Given the quantitative design of these studies, the extant literature has not been able to explain why Asian American experiences of social support can contribute to greater rather than lesser distress. Our findings fill a gap in the literature to explain how Asian Americans experience victimization from their support networks, given the focus on impact rather than intent of social support.

Without ever asking participants to provide details about the racial background of their support network, our participants spontaneously offered the racial identities of their support systems. Specifically, White partners/spouses emerged consistently in our analyses as being unable to provide the kind of support that our participants needed. Additionally, when our participants described their experiences of failed social support, they provided racial context to distinguish the kinds of support offered by White friends, Asian American friends, and non-Asian People of Color friends. Our findings underscore the importance of assessing the racial identities of participant social support networks, especially in the context of seeking support for racial victimization.

The harmful consequences of social support from White people were noteworthy in this study, and highlighted the critical role of White partners/spouses and White friends. Research on the protective effect of spousal support has paid little attention to spousal racial identity and its implications. This can explain why, in secondary archival data analysis of national data, Rollock and Lui (18) only saw the buffering effects of spousal support against distress associated with unfair treatment, but not racial discrimination. In contrast, qualitative work by Lowe et al. (34) has underscored the critical role of White partners/spouses in exacerbating the racial trauma of those seeking support after racist victimization. Additionally, in examining the role of friends, Mossakowski and Zhang (15) found little value for friend support. In other studies (22–25), the social support of romantic partners, friends, and family members have all been studied together, rather than independently assessed for their unique contributions. The descriptive nature of our study findings sheds light on the unique role of each type of confidante and the harm they can inflict on Asian Americans seeking support in the aftermath of racism. Clearly, there is a need to recognize the differential harm that can be perpetrated by one's support system.

Qualitative research by Lowe et al. (34) has shown that People of Color experience secondary injuries when confiding in others about racism. Even more, in their study on racism, trauma, and coping, the authors concluded that “what felt most injurious [to the participants] was when a confidant would minimize participant experiences, dismiss the significance and impact of a racist event on them, question their interpretation of the event, and invalidate their thoughts and feelings…” (p. 194). The themes in our study build on this theme to delineate the range of “injuries” committed by members of their support system. Given that our participants were already victims of racism, we underscore the work of Lowe et al. (34) to attend to trauma-informed responses to support victims of racial discrimination.

An important finding in this study is that Asian Americans are not immune to perpetrating additional racial trauma toward one another. American families and friends added to the “injury” of Asian American victims of racism. Gee et al. (35) found that family members contribute to being a barrier in Asian American help-seeking and mental health service utilization. In addition, other studies have found that family factors (e.g., conflict and cohesion) and generational status affected Asian American use of mental health services (36, 37). Thus, our findings are consistent with our participants' recommendations to improve social support by de-stigmatizing mental health in ways that attend to intergenerational differences within the Asian American community. While it is not novel to conclude that stigma, beliefs about the causes of mental illness, and unfamiliarity with mental health services are cultural barriers for Asian Americans to utilize mental health services (38). However, because Asian American families and friends can add secondary injury to those needing social support, our findings underscore the need to enhance Asian American mental health literacy to better support the multiple generations of Asian Americans suffering from racial trauma.

The revelatory nature of our study is an added contribution to social support literature. It is important note that our participants participated in this study during a time when COVID-19 was evolving from an endemic to a pandemic, and parts of the U.S. were enacting policies such as social distancing, working from home, and sheltering-in-place. As such, the revelatory nature of our study may have successfully captured the most crucial people in their support network, specifically, their inner circle. As a result of COVID-19, our participants likely did not have opportunities to conveniently run into acquaintances given that they were not able to leave the house. By being restricted to their homes, the people they solicited for support were likely to have been their most-trusted confidants. Consequently, the revelatory nature of our study likely allowed us to identify those individuals who constitute the most important roles in their support network: romantic partners/spouses, parents and siblings, friends, and “others,” such as colleagues, neighbors, and their online communities.

Regarding future scholarship examining the mediating and moderating effects on social support on racism and Asian American wellbeing, our findings suggest that the frameworks used to study social support must be expanded to account for systemic influences. A focus on individual coping may be overly narrow, and more scholarship is needed to examine the quality of social support. Additionally, despite the growing body of scholarship on Asian American stress and coping (32, 39), it is important to recognize that the stress-coping framework seemingly assumes individual-level responsibility by focusing on an individual's capability to cope with and heal from anti-Asian racism. That is, the stress-coping framework focuses on individual capability—namely, the ability for an individual to cope with racism depending on whether they are able or unable to access resources to respond to harmful events (40). However, coping should not be conceptualized as an individual-level experience, but instead, one that has to do with cultural and environmental supports and resources. Noh and Kaspar (41) have cautioned that it is important to recognize individual access to capital such as social resources and educational capital. In the current study, our findings underscore the importance of adequately supporting Asian Americans by placing the responsibility of racism on the larger society and not just to Asian Americans or even their immediate social support system. To meaningfully improve Asian American social support, all of the recommendations provided by our participants target societal accountability rather than individual- or even community-level responsibility. Supporting Asian Americans therefore requires de-stigmatizing mental health, increasing representation of Asian Americans in media and political spheres, making Asian American history part of the academic curriculum, and acknowledging anti-Asian racism as a long-standing part of U.S. history.

As an example, the legal requirement in California to include ethnic studies in high schools resembles one way in which public education can be used to dismantle white supremacy. Additional forms of institutional and structural support are needed to combat racism, especially given the 2021–2022 backlash to racial justice policies. As of 2022, 28 states have introduced or taken steps to restrict the teaching of Critical Race Theory or limited how teachers can discuss racism, with 11 states enacting these bans through legislation or other avenues (42). Thus, while our participants underscored the importance of teaching Asian American history for empowerment (43), their message also highlights the need for societal interventions to intervene and also prevent racism: through public education and media (44), increased political involvement (45), and cross- racial and cross-ethnic forms of coalition-building (46) *via* ethnic studies. With regard to Asian American health inequities, this also means investing in clinical research with Asian Americans, given the underrepresentation of Asian American funding in the National Institutes of Health research budget (47).

That participants underscore the need for collective, societal learning underscores the need for all communities and not just White societal members to confront their stereotypes and assumptions about Asian Americans. This need is particularly urgent given the data that has shown that one-third of Asian Americans nationwide fear threats, physical attacks, and violence; and that approximately 80% perceive violence against them to be increasing (48). Quantitative findings by Zhang et al. (49) have also shown that Asian Americans experience a unique risk to violence. Based on their analysis of 12 years of data from the National Incident-Based Reporting System, they compared the nature of anti-Asian hate crimes from anti-Black and anti-Latinx hate crimes. The authors found that anti-Asian hate crimes were more likely than the hate crimes against Black and Latinx communities to be committed by POC perpetrators (49). They speculated resentment and animosity surrounding Asian American stereotypes to be possible reasons that Asian Americans are targeted. As such, consistent with our participants' recommendations, offering support to Asian Americans means enlisting the help of all societal members, including Asian Americans, White Americans, and other communities of Color to learn about Asian American experiences of racism.

In short, our findings indicate that social support for Asian Americans victims of racism should not be assumed to be unequivocally beneficial. Well-intentioned support from loved ones can actually yield additional harm and distress. By designing a study that examined impact instead of the intent of social support, our findings suggest that Asian Americans may be wise in under-utilizing support as well as underreporting experiences of racist victimization. For example, Asian American under-reporting has been acknowledged in a number of contexts, from anti-Asian violence and hate crimes (50) to experiences of discrimination and isolation (51), economic hardship (52), and Asian American mental health needs (53, 54). The invisibility of anti-Asian violence is not new, and has been referred to as “the silent dilemma” due to public unawareness, apathy, and resistance toward recognizing hate crimes conducted against Asian Americans, as well as stereotypes of Asian Americans being a model minority (50). As such, rather than encouraging Asian Americans to speak out, speak up, or to ask for help, our research findings empirically demonstrate the unintended effects of social support in which Asian Americans may experience additional harm and injury when seeking support from trusted, informal support networks. As such, rather than focus on Asian American coping as points of interventions, it is up to society to invest in the necessary resources to become better forms of social support for Asian American victims of racism.

## Limitations and implications

This study is part of a larger investigation examining anti-Asian racism during COVID-19. As such, this paper emerged from a larger qualitative case study that was not designed to specifically examine the social support of Asian Americans. Additionally, we did not collect demographic data on gender, ethnic group, and generational status, which limits us from having a more nuanced understanding of our participants' experiences. Recent data from the Stop AAPI Hate advocacy report (31) found that 68% of the individuals reporting hate incidents identify as Chinese, Korean, or Filipinx. Additionally, one of the key findings in the report was that Asian Americans identifying as female, non-binary, LGBTQIA+ and older adults experienced hate incidents that targeted them for more than one of their identities at once. Thus, the intersections of sex, gender, sexual orientation, and age can be especially important for understanding the marginalization and risks of some Asian Americans subgroups. Our study was not able to account for analyses that address intersectionality because we did not collect these kinds of demographic information. Future research would benefit from asking these key demographics, as well as gathering background information such as education level, income level, and immigration status, particularly in understanding Asian American needs for social support.

Our study has important implications for providing social support for Asian Americans in response to racism. Beyond the concrete recommendations provided by our participants, mental health care providers can draw on these findings to provide affinity spaces for Asian Americans, such as group therapy. The benefits of technology and social media also allow for opportunities to facilitate a sense of community even in settings where there may be fewer Asian Americans and fewer cultural resources for Asian Americans. Administrators in school, community, organizational, and corporate settings can also benefit from recognizing the unique isolation that Asian Americans experience, and therefore, offer programs and initiatives that generate greater community and belonging for Asian Americans. Learning and supporting the experiences of Asian Americans must be a collective, societal effort rather than a responsibility placed on victims of racism and their immediate social support networks. For example, May is considered Asian American Pacific Islander heritage month, and it is a time when the country is supposed to acknowledge, celebrate, and learn about Asian American history, media, politics, and leadership positions across different disciplines. Relatedly, the month of May is also mental health awareness month, and provides the opportunity to specifically recognize the culture-specific stigmas and barriers that impede as well as facilitate Asian American mental health needs as well as resilience. As evidenced in our participants' responses, it is critical to understand the intergenerational and racial trauma that Asian American families experience, given the long-standing marginalization of Asian Americans in U.S. history and presently. Supporting Asian Americans therefore requires support systems to be implemented at the societal and structural level, in the form of prevention and intervention services, and with urgency as well as recognition of the enduring nature of anti-Asian racism throughout this country's history.

## Conclusion

Our study descriptively captures the types of inadequate social support received by Asian Americans during the first few months of the COVID-19 pandemic. While social support is, in theory, meant to be supportive, our findings showcased a range of injurious responses. Our findings add to the quantitative work that calls for more scholarship examining the nature and quality of social support. Additionally, our study highlights the need to understand the subjective experiences of those receiving support, so that research on social support can be defined by participants rather than by pre-determined definitions. Furthermore, social support must be understood as a collective responsibility, one that is implemented at the societal level when it comes to preventing, intervening, and supporting Asian Americans from anti-Asian racism.

## Data availability statement

The raw data supporting the conclusions of this article will be made available by the authors, without undue reservation.

## Ethics statement

The studies involving human participants were reviewed and approved by Santa Clara University Institutional Review Board. The participants provided their written informed consent to participate in this study.

## Author contributions

SCW: study design and data collection. SCW and BMCS: data analysis, manuscript writing, and intellectual content. Both authors contributed to the article and approved the submitted version.

## Conflict of interest

The authors declare that the research was conducted in the absence of any commercial or financial relationships that could be construed as a potential conflict of interest.

## Publisher's note

All claims expressed in this article are solely those of the authors and do not necessarily represent those of their affiliated organizations, or those of the publisher, the editors and the reviewers. Any product that may be evaluated in this article, or claim that may be made by its manufacturer, is not guaranteed or endorsed by the publisher.

## References

[1] Chen JA Zhang E Liu CH. Potential impact of COVID-19-related racial discrimination on the health of Asian Americans. Am J Public Health. (2020) 110:1624–7. 10.2105/AJPH.2020.305858 32941063 PMC7542280

[2] Pew Research Center. Many Black Asian Americans Say They Have Experienced Discrimination Amid the COVID-19 Outbreak. Washington, DC. Pew Research Center (US) (2020). Available online at: https://www.pewresearch.org/social-trends/2020/07/01/many-black-and-asian-americans-say-they-have-experienced-discrimination-amid-the-covid-19-outbreak/

[3] Tessler H Choi M Kao G. The anxiety of being Asian American: hate crimes and negative biases during the COVID-19 pandemic. Am J Crim Justice. (2020) 45:636–46. 10.1007/s12103-020-09541-5 32837158 PMC7286555

[4] Donaghue E. Surging anti-Asian hate crimes being tracked during coronavirus pandemic: “Things are getting very physical”. CBS News. (2020). Available online at: https://www.cbsnews.com/news/coronavirus-pandemic-anti-asian-hate-crimes-tracking/

[5] Stop AAPI Hate. Stop AAPI Hate National Report 3.19.20 - 8.5.20. Stop AAPI Hate (US) (2020). Available online at: https://stopaapihate.org/wp-content/uploads/2021/04/Stop-AAPI-Hate-Report-National-200805.pdf

[6] Heaney CA Israel BA. Social networks and social support. In:Glanz K Rimer BK Viswanath K, editors. Health Behavior and Health Education: Theory, Research, and Practice. San Francisco, CA: Josey-Bass (2008). p. 189–207.

[7] US Department of Health and Human Services. Mental Health: Culture, Race, and Ethnicity - A Supplement to Mental Health, a Report of the Surgeon General: Executive Summary. Rockville, MD: Substance Abuse and Mental Health Services Administration (2001).

[8] Spencer MS Chen J Gee GC Fabian CG Takeuchi DT. Discrimination and mental health-related service use in a national study of Asian Americans. Am J Public Health. (2010) 100:2410–7. 10.2105/AJPH.2009.176321 20299649 PMC2978178

[9] Cho H Kim I Velez-Ortiz D. Factors associated with mental health service use among Latino and Asian Americans. Community Ment Health J. (2014) 50:960–7. 10.1007/s10597-014-9719-6 24659219

[10] Kim PY Kendall DL Chang ES. Emotional self-control, interpersonal shame, and racism as predictors of help-seeking attitudes among Asian Americans: an application of the intrapersonal-interpersonal-sociocultural framework. Asian Am J Psychol. (2016) 7:15–24. 10.1037/aap0000032

[11] Leong FT Lau AS. Barriers to providing effective mental health services to Asian Americans. Ment Health Serv Res. (2001) 3:201–14. 10.1023/A:1013177014788 11859966

[12] Tran B. Understanding and addressing the stigma in mental health within the Asian and Asian-American culture. In:Canfield BA Cunningham HA, editors. Deconstructing Stigma in Mental Health. Hershey, PA: IGI Global (2018). p. 70–107.

[13] Bauer AM Chen C Alegria M. English language proficiency and mental health service use among Latino and Asian Americans with mental disorders. Med Care. (2010) 41:1097–104. 10.1097/MLR.0b013e3181f80749 21063226 PMC3135417

[14] Chou C. Critique on the notion of model minority: an alternative racism to Asian American? Asian Ethn. (2008) 9:219–29. 10.1080/14631360802349239

[15] Mossakowski KN Zhang W. Does social support buffer the stress of discrimination and reduce psychological distress among Asian Americans? Soc Psychol Q. (2014) 77:273–95. 10.1177/0190272514534271

[16] Kim I. The role of critical ethnic awareness and social support in the discrimination-depression relationship among Asian Americans: path analysis. Cultur Divers Ethnic Minor Psychol. (2014) 20:52–60. 10.1037/a0034529 24491128

[17] Kim H Epstein NB. Racism, stress and health in Asian Americans: a structural equation analysis of mediation and social support group differences. Stress Health. (2020) 37:103–15. 10.1002/smi.2979 32790912

[18] Rollock D Lui PP. Do spouses matter? Discrimination, social support, and psychological distress among Asian Americans. Cultur Divers Ethnic Minor Psychol. (2016) 22:47–57. 10.1037/cdp0000045 25867552

[19] Chae DH Lee S Lincoln KD Ihara ES. Discrimination, family relationships, and major depression among Asian Americans. J Immigr Minor Health. (2011) 14:361–70. 10.1007/s10903-011-9548-4 22083344 PMC3501208

[20] Wei M Yeh CJ Chao RC-L Carrera S Su JC. Family support, self-esteem, and perceived racial discrimination among Asian American male college students. J Couns Psychol. (2013) 60:453–61. 10.1037/a0032344 23544839

[21] Singh S McBride K Kak V. Role of social support in examining acculturative stress and psychological distress among Asian American immigrants and three sub-groups: results from NLAAS. J Immigr Minor Health. (2015) 17:1597–606. 10.1007/s10903-015-0213-1 25910620

[22] Chung H Epstein NB. Perceived racial discrimination, acculturative stress, and psychological distress among Asian immigrants: the moderating effects of support and interpersonal strain from a partner. Int J Intercult Relat. (2014) 42:129–39. 10.1016/j.ijintrel.2014.04.003

[23] Kwon S. Perceived discrimination, family and spousal relationships, and psychological distress among Asian Americans: testing mediation and moderation effects. J Soc Sci. (2020) 57:26–38. 10.1016/j.soscij.2019.01.001

[24] Nadimpalli SB Kanaya AM McDade TW Kandula NR. Self-reported discrimination and mental health among Asian Indians: cultural beliefs and coping style as moderators. Asian Am J Psychol. (2016) 7:185–94. 10.1037/aap0000037 27668066 PMC5030840

[25] Alemi Q Siddiq H Baek K Sana H Stempel C Aziz N. Effect of perceived discrimination on depressive symptoms in 1st- and 2nd-generation Afghan-Americans. J Prim Prev. (2017) 38:613–26. 10.1007/s10935-017-0492-5 29071488

[26] Gee GC Chen J Spencer MS See S Kuester OA Tran D. Social support as a buffer for perceived unfair treatment among Filipino Americans: differences between San Francisco and Honolulu. Am J Public Health. (2006) 96:677–84. 10.2105/AJPH.2004.060442 16507727 PMC1470535

[27] Wang SC Santos BMC. “Go back to China with your (expletive) virus”: a revelatory case study of anti-Asian racism during COVID-19. Asian Am J Psychology. (2022). 10.1037/aap0000287

[28] Yin RK. Case Study Research: Design and Methods. 4th ed. Thousand Oaks, CA: Sage (2009).

[29] Braun V Clarke V. Using thematic analysis in psychology. Qual Res Psychol. (2006) 3:77–101. 10.1191/1478088706qp063oa

[30] Creswell JW Miller DL. Determining validity in qualitative inquiry. Theory Pract. (2000) 39:124–30. 10.1207/s15430421tip3903_2

[31] Stop AAPI Hate. Two Years Thousands of Voices: What Community-Generated Data Tells Us About Anti-AAPI Hate. Stop AAPI Hate (US) (2022). Available online at: https://stopaapihate.org/wp-content/uploads/2022/07/Stop-AAPI-Hate-Year-2-Report.pdf

[32] Liang CTH Alvarez AN Juang LP Liang MX. The role of coping in the relationship between perceived racism and racism-related stress for Asian Americans: gender differences. J Couns Psychol. (2007) 54:132–41. 10.1037/0022-0167.54.2.132

[33] Alvarez AN Juang LP. Filipino Americans and racism: a multiple mediation model of coping. J Couns Psychol. (2010) 57:132–14. 10.1037/a0019091 21133568

[34] Lowe SM Okubo Y Reilly MF. A qualitative inquiry into racism, trauma, and coping: implications for supporting victims of racism. Prof Psych Res Pr. (2012) 43:190–8. 10.1037/a0026501

[35] Gee CB Khera GS Poblete AT Kim B Buchwach SY. Barriers to mental health service use in Asian American and European American college students. Asian Am J Psychol. (2020) 11:98–107. 10.1037/aap0000178

[36] Chang J Natsuaki MN Chen C-N. The importance of family factors and generation status: mental health service use among Latino and Asian Americans. Cultur Divers Ethnic Minor Psychol. (2013) 19:236–47. 10.1037/a0032901 23875849

[37] Ta VM Holck P Gee GC. Generational status and family cohesion effects on the receipt of mental health services among Asian Americans: findings from the National Latino and Asian American Study. Am J Public Health. (2010) 100:115–21. 10.2105/AJPH.2009.160762 19910344 PMC2791260

[38] Tung W-C. Cultural barriers to mental health services among Asian Americans. Home Health Care Manag Pract. (2011) 23:303–5. 10.1177/1084822311401857

[39] Lee RM Liu HTT. Coping with intergenerational family conflict: comparison of Asian American, Hispanic, and European American college students. J Couns Psychol. (2001) 48:410–9. 10.1037/0022-0167.48.4.410

[40] Lazarus RS Folkman S. Stress, Appraisal, and Coping. New York, NY: Springer Publishing Company (1984).

[41] Noh S Kaspar V. Perceived discrimination and depression: moderating effects of coping, acculturation, and ethnic support. Am J Public Health. (2003) 93:232–8. 10.2105/AJPH.93.2.232 12554575 PMC1447722

[42] Rennie LB. Federal and State Racism Education Policy Landscape. Washington, DC: Advocacy Office, American Psychological Association (2021).

[43] Trieu MM Lee HC. Asian Americans and internalized racial oppression: identified, reproduced, and dismantled. Sociol Race Ethnic. (2017) 4:67–82. 10.1177/2332649217725757

[44] Helms JE. Taking action against racism in a post-racism era. Couns Psychol. (2015) 43:138–45. 10.1177/0011000014564250

[45] Chou RS Feagin JR. The myth of the model minority: Asian Americans facing racism. Boulder, CO: Paradigm Publishers (2015).

[46] Harrell SP. A multidimensional conceptualization of racism-related stress: implications for the well-being of people of color. Am J Orthopsychiatry. (2000) 70:42–57. 10.1037/h0087722 10702849

[47] Doan LN Takata Y Sakuma KK Irvin VL. Trends in clinical research including Asian American, Native Hawaiian, and Pacific Islander participants funded by the US National Institutes of Health, 1992 to 2018. JAMA Netw Open. (2019) 2:e197432. 10.1001/jamanetworkopen.2019.7432 31339543 PMC6659145

[48] Pew Research Center. One-Third of Asian Americans Fear Threats, Physical Attacks Most Say Violence Against Them Is Rising. Washington, DC: Pew Research Center (US) (2021). Available online at https://www.pewresearch.org/fact-tank/2021/04/21/one-third-of-asian-americans-fear-threats-physical-attacks-and-most-say-violence-against-them-is-rising/

[49] Zhang Y Zhang L Benton F. Hate crimes against Asian Americans. Am J Crim Justice. (2021) 47:441–61. 10.1007/s12103-020-09602-9 33437139 PMC7790522

[50] Kia N. Asian Americans' experiences of “race” and racism. In:Feagin J Vera H, editors. Handbooks of the Sociology of Racial and Ethnic Relations. Boston, MA: Springer (2007). p. 131–44.

[51] Lee S Juon HS Martinez G Hsu CE Robinson ES Bawa J. Model minority at risk: expressed needs of mental health by Asian American young adults. J Comm Health. (2009) 34:144–52. 10.1007/s10900-008-9137-1 18931893 PMC3296234

[52] Ishii-Kuntz M Gomel JN Tinsley BJ Parke RD. Economic hardship and adaptation among Asian American families. J Fam Issues. (2010) 31:407–20. 10.1177/0192513X09351271

[53] Leong FT Lau AS. Barriers to providing effective mental health services to Asian Americans. Mental Health Servs Rsch. (2001) 3:215–23. 10.1023/a:1013177014788 11859966

[54] Ling A Okazaki S Tu MC Kim JJ. Challenges in meeting the mental health needs of urban Asian American adolescents: service providers' perspectives. Race Soc Probs. (2014) 6:25–37. 10.1037/e534172014-001

